# Supplementary material for: Spatiotemporal proteomics reveals the biosynthetic lysosomal membrane protein interactome in neurons
Source: Nat Commun. 2024 Dec 30;15:10829. doi: 10.1038/s41467-024-55052-w (PMC11868546; doi:10.1038/s41467-024-55052-w)
Supplement: Supplementary file 1 — Supplementary Information [file 41467_2024_55052_MOESM1_ESM.pdf]

Supplementary Information

Spatiotemporal proteomics reveals the biosynthetic lysosomal membrane protein interactome in neurons

Chun Hei Li[1], Noortje Kersten[1], Nazmiye Özkan[1], Dan T. M. Nguyen[1], Max Koppers[1,2], Harm Post[3], Maarten Altelaar[3], Ginny G. Farias[1,4 *]

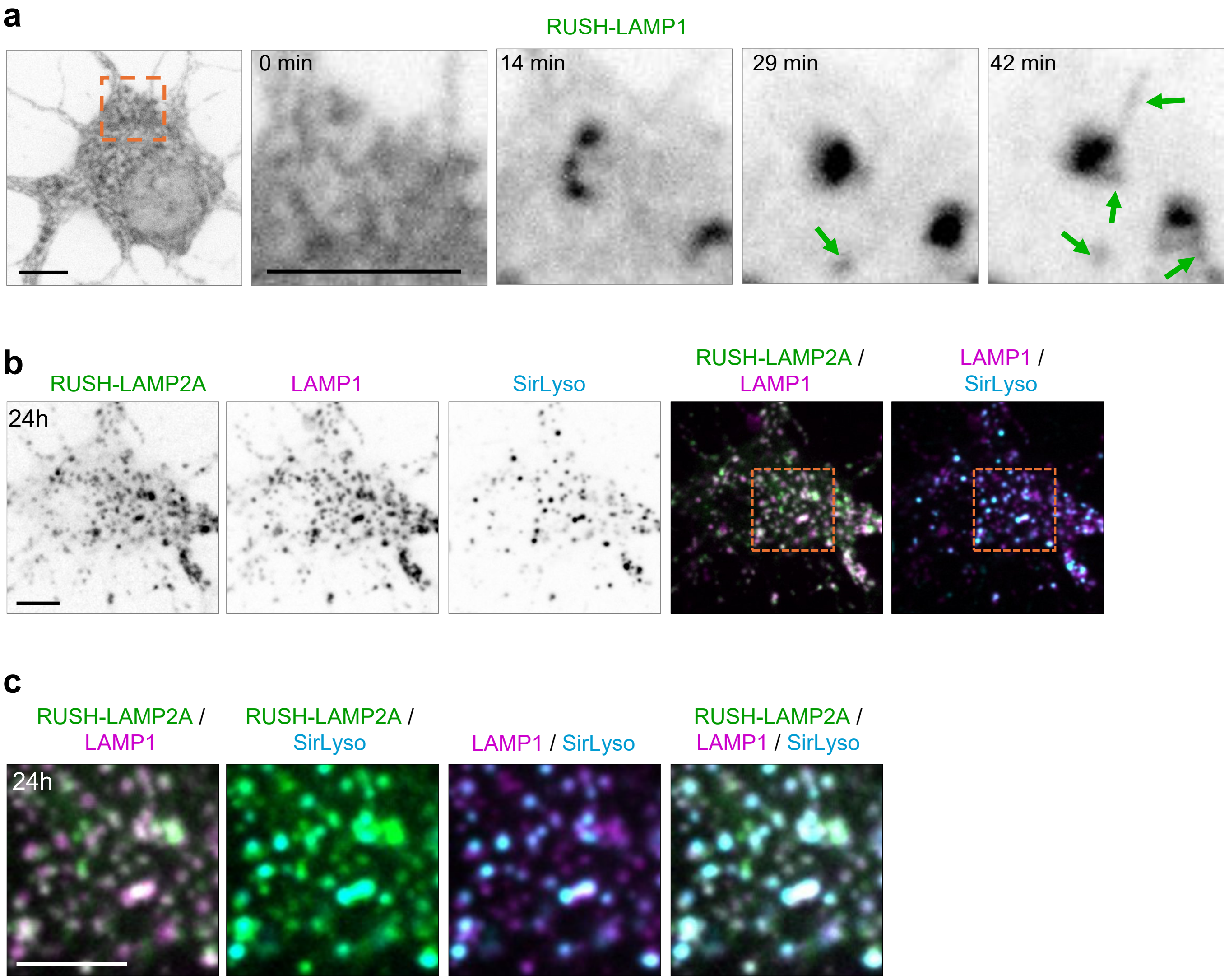
a
RUSH-LAMP1
0 min
14 min
29 min
42 min
b
RUSH-LAMP2A
LAMP1
SirLyso
RUSH-LAMP2A / LAMP1
LAMP1 / SirLyso
24h
c
RUSH-LAMP2A / LAMP1
RUSH-LAMP2A / SirLyso
LAMP1 / SirLyso
RUSH-LAMP2A / LAMP1 / SirLyso
24h

Supplementary Fig. 1 – Related to Fig. 1: Biosynthetic LAMP release dynamics after biotin addition and their lysosomal distribution 24h after release.

a, Representative still images from live hippocampal neurons expressing RUSH-LAMP1-mScarlet-I and imaged in a confocal spinning disk immediately after biotin addition every 1 min for 1h. Scale bar, 5 µm. **b-c,** Representative still images from neurons co-expressing RUSH-LAMP2A-mNG and LAMP1-RFP (to visualize its steady state lysosomal distribution) and live-labeled with SirLyso 20 min prior to imaging to visualize mature lysosomes 24h after biotin addition. Selected magnified region in orange boxes, in c. Scale bar, 5 µm. Representative images were repeated in at least 3 independent experiments.

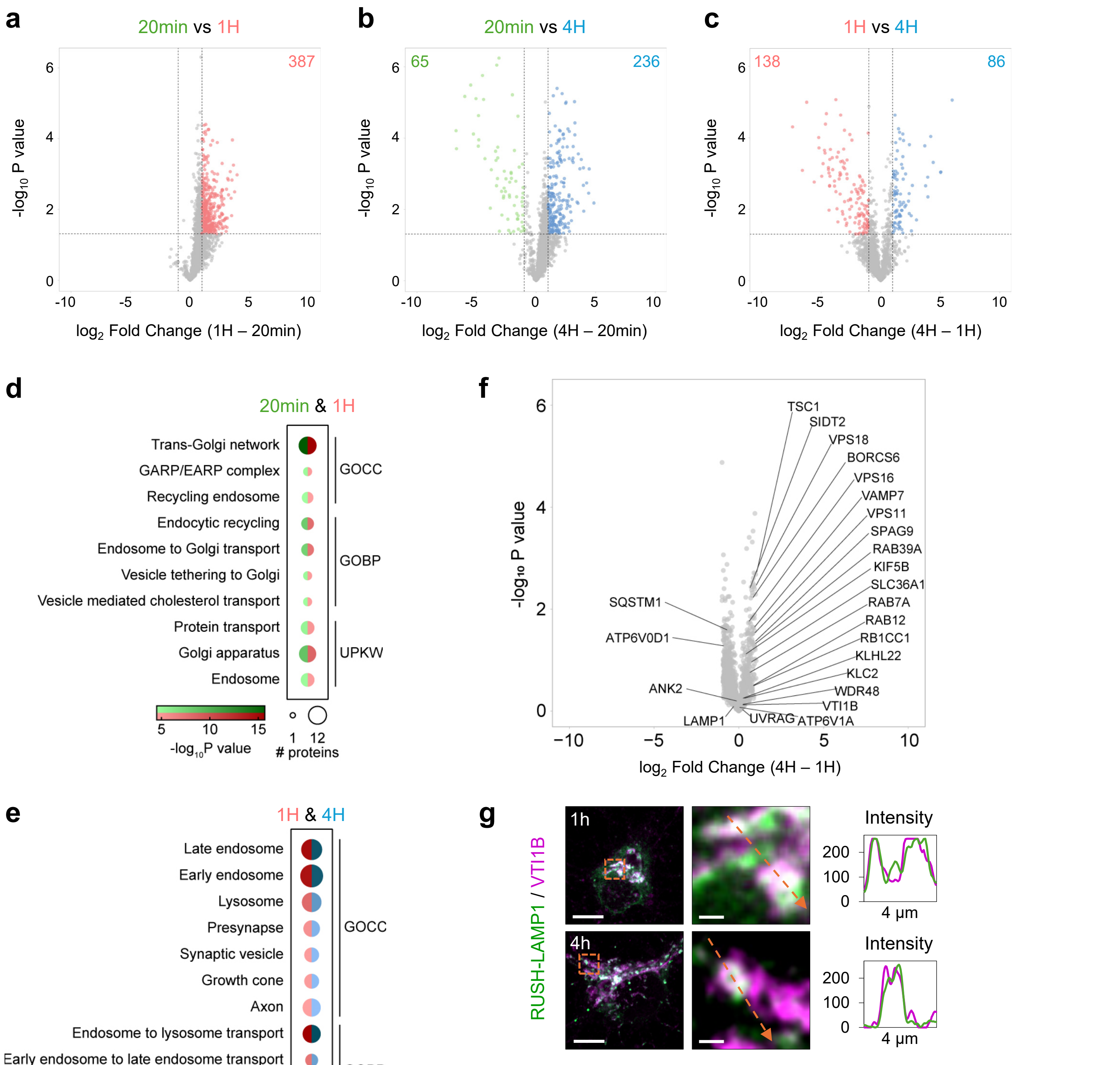

h

Selected 4H Unique Hits

Protein Name	Total Peptide Count
AP5B1	32
ATG2B	19
ATP13A2	19
BLOC1S1	12
SNAPIN	14
DENND10	10
TBC1D9	26
COMMD2	9
VPS28	13
WASHC4	25

Supplementary Fig. 2 – Related to Figs. 2 & 3: POTATOMap reveals distinct interactome over-time.

a-c, Volcano plots of proteins identified by MS analysis, plotted according to differential enrichment between time points (**a**, 1h/20min; **b**, 4h/20min; **c**, 4h/1h; N = 2 independent biological samples with 2 technical repeats per sample). Horizontal dashed line indicates significance threshold ($p<0.05$); vertical dashed lines indicate fold change cut-off ($\log_2$ Fold Change ≥ 1 or $\log_2$ Fold Change ≤ -1) (two-sided t-test, no multiple comparison test was used). The number of proteins considered significantly changing are indicated in the top left and right corner of each plot. **d**, **e**, DAVID analysis on Gene Ontology (GO) terms enriched among the proteins shared between time points (d, 20min and 1h; e, 1h and 4h) (FDR < 0.01). Dot size represents the number of proteins, dot color represents respective time point and term enrichment (p value < 0.05). GOCC, cellular compartment; GOBP, biological process; GOMF, molecular function; UPKW, Uniprot keyword. **f**, Partial illustration of the volcano plot shown (**c**) including only the non-changing proteins between 1h and 4h. **g**, Confocal images of the soma of neurons expressing RUSH-LAMP1-V5, fixed 1h or 4h post-release and stained for VTI1B. Orange boxes indicate magnified regions. Orange arrows indicate the region used to generate the intensity profile graphs. Scale bar, 10 µm, scale bar magnified images, 1 µm. **h**, table showing selected 4h unique hits and their respective total peptide counts. Representative images were repeated in 3 independent experiments. Source data are provided as a Source Data file (See Source Data 1 and 2 for detailed analysis).

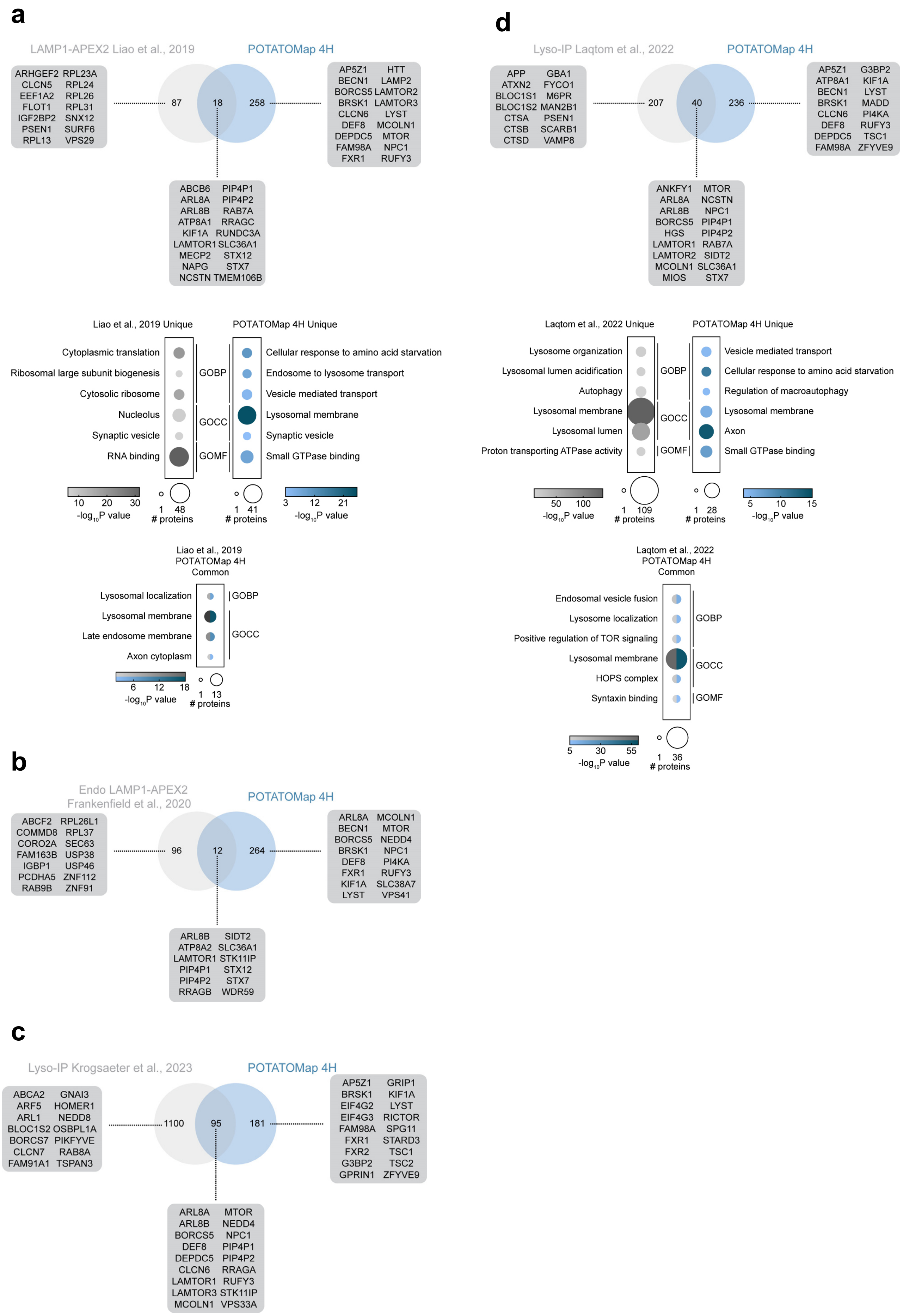

a
LAMP1-APEX2 Liao et al., 2019
POTATOMap 4H
87
18
258
ARHGEF2 RPL23A
CLCN5 RPL24
EEF1A2 RPL26
FLOT1 RPL31
IGF2BP2 SNX12
PSEN1 SURF6
RPL13 VPS29
AP5Z1 HTT
BECN1 LAMP2
BORCS5 LAMTOR2
BRSK1 LAMTOR3
CLCN6 LYST
DEF8 MCOLN1
DEPDC5 MTOR
FAM98A NPC1
FXR1 RUFY3
ABCB6 PIP4P1
ARL8A PIP4P2
ARL8B RAB7A
ATP8A1 RRAGC
KIF1A RUNDC3A
LAMTOR1 SLC36A1
MECP2 STX12
NAPG STX7
NCSTN TMEM106B
Liao et al., 2019 Unique
POTATOMap 4H Unique
Cytoplasmic translation
Ribosomal large subunit biogenesis
Cytosolic ribosome
Nucleolus
Synaptic vesicle
RNA binding
GOBP
GOCC
GOMF
Cellular response to amino acid starvation
Endosome to lysosome transport
Vesicle mediated transport
Lysosomal membrane
Synaptic vesicle
Small GTPase binding
10 20 30
-log10P value
1 48
proteins
1 41
proteins
3 12 21
-log10P value
Liao et al., 2019
POTATOMap 4H
Common
Lysosomal localization
Lysosomal membrane
Late endosome membrane
Axon cytoplasm
GOBP
GOCC
6 12 18
-log10P value
1 13
proteins
b
Endo LAMP1-APEX2
Frankenfield et al., 2020
POTATOMap 4H
96
12
264
ABCF2 RPL26L1
COMMD8 RPL37
CORO2A SEC63
FAM163B USP38
IGBP1 USP46
PCDHA5 ZNF112
RAB9B ZNF91
ARL8A MCOLN1
BECN1 MTOR
BORCS5 NEDD4
BRSK1 NPC1
DEF8 PI4KA
FXR1 RUFY3
KIF1A SLC38A7
LYST VPS41
ARL8B SIDT2
ATP8A2 SLC36A1
LAMTOR1 STK11IP
PIP4P1 STX12
PIP4P2 STX7
RRAGB WDR59
c
Lyso-IP Krogsaeter et al., 2023
POTATOMap 4H
1100
95
181
ABCA2 GNAI3
ARF5 HOMER1
ARL1 NEDD8
BLOC1S2 OSBPL1A
BORCS7 PIKFYVE
CLCN7 RAB8A
FAM91A1 TSPAN3
AP5Z1 GRIP1
BRSK1 KIF1A
EIF4G2 LYST
EIF4G3 RICTOR
FAM98A SPG11
FXR1 STARD3
FXR2 TSC1
G3BP2 TSC2
GPRIN1 ZFYVE9
ARL8A MTOR
ARL8B NEDD4
BORCS5 NPC1
DEF8 PIP4P1
DEPDC5 PIP4P2
CLCN6 RRAGA
LAMTOR1 RUFY3
LAMTOR3 STK11IP
MCOLN1 VPS33A
d
Lyso-IP Laqtom et al., 2022
POTATOMap 4H
207
40
236
APP GBA1
ATXN2 FYCO1
BLOC1S1 M6PR
BLOC1S2 MAN2B1
CTSA PSEN1
CTSB SCARB1
CTSD VAMP8
AP5Z1 G3BP2
ATP8A1 KIF1A
BECN1 LYST
BRSK1 MADD
CLCN6 PI4KA
DEF8 RUFY3
DEPDC5 TSC1
FAM98A ZFYVE9
ANKFY1 MTOR
ARL8A NCSTN
ARL8B NPC1
BORCS5 PIP4P1
HGS PIP4P2
LAMTOR1 RAB7A
LAMTOR2 SIDT2
MCOLN1 SLC36A1
MIOS STX7
Laqtom et al., 2022 Unique
POTATOMap 4H Unique
Lysosome organization
Lysosomal lumen acidification
Autophagy
Lysosomal membrane
Lysosomal lumen
Proton transporting ATPase activity
GOBP
GOCC
GOMF
Vesicle mediated transport
Cellular response to amino acid starvation
Regulation of macroautophagy
Lysosomal membrane
Axon
Small GTPase binding
50 100
-log10P value
1 109
proteins
1 28
proteins
5 10 15
-log10P value
Laqtom et al., 2022
POTATOMap 4H
Common
Endosomal vesicle fusion
Lysosome localization
Positive regulation of TOR signaling
Lysosomal membrane
HOPS complex
Syntaxin binding
GOBP
GOCC
GOMF
5 30 55
-log10P value
1 36
proteins

Supplementary Fig. 3 – Related to Fig. 2: POTATOMap at 4h identifies key lysosomal proteins and new candidates.

a-d, Venn diagrams comparing POTATOMap 4h lysosomal timepoint to different lyso-proteomics approaches. **a**, Comparison of POTATOMap 4h to LAMP1-APEX2 in iPSC-derived neurons from Liao et al., 2019[27]. **b**, Comparison of POTATOMap 4h to endogenously knocked-in LAMP1-APEX2 in iPSC-derived neurons from Frankenfield et al., 2023[31]. **c**, Comparison of POTATOMap 4h to neuro-2a cells Lyso-IP from Krogsaeter et al.,2023[32] **d**, Comparison of POTATOMap 4h to in-vivo mouse Lyso-IP from Laqtom et al., 2022[25]. Known lysosomal proteins were highlighted in overlapping candidates. Selected unique proteins related to endolysosomes and other biological processes were highlighted for each dataset. Gene Ontology (GO) terms enriched for each dataset comparison (a, d, p value < 0.05, FDR < 0.01). Dot size represents the number of different proteins, dot color represents respective time point and term enrichment (p value < 0.05). GOCC, cellular compartment; GOBP, biological process; GOMF, molecular function; UPKW, Uniprot keyword. Source data are provided as a Source Data file (See Source Data 1 for detailed analysis).

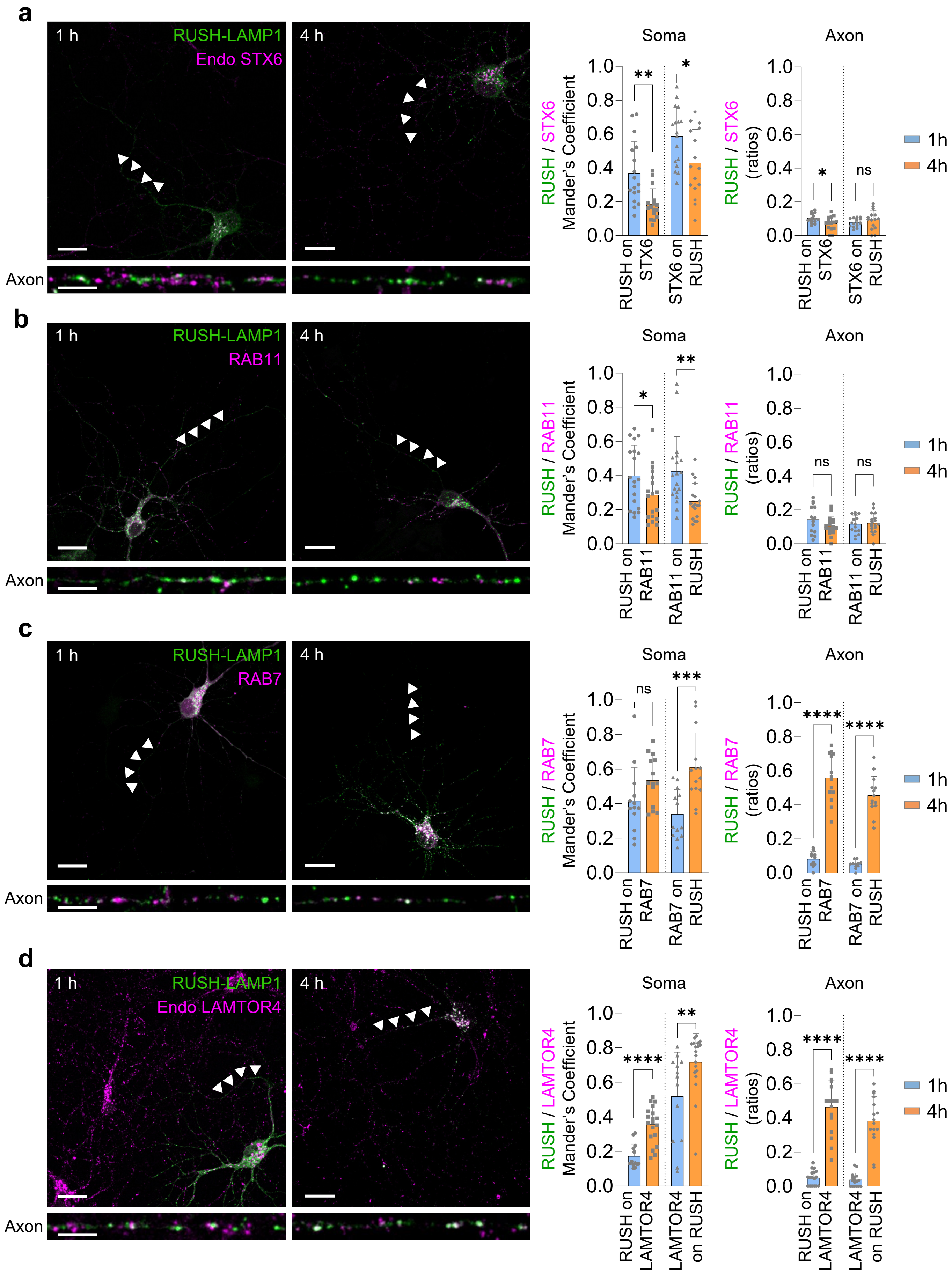
a
b
c
d
1 h
4 h
RUSH-LAMP1
Endo STX6
RAB11
RAB7
Endo LAMTOR4
Axon
Soma
Axon
Mander's Coefficient
RUSH / STX6
RUSH / STX6 (ratios)
RUSH on STX6
STX6 on RUSH
RUSH / RAB11
RUSH / RAB11 (ratios)
RUSH on RAB11
RAB11 on RUSH
RUSH / RAB7
RUSH / RAB7 (ratios)
RUSH on RAB7
RAB7 on RUSH
RUSH / LAMTOR4
RUSH / LAMTOR4 (ratios)
RUSH on LAMTOR4
LAMTOR4 on RUSH
ns
1h
4h

Supplementary Fig. 4 – Related to Fig. 3: Differential biosynthetic LAMP distribution with different POTATOMap candidates over time.

a-d, left, Representative images of RUSH-LAMP1 after 1 and 4h of biotin in soma and axon co-labelled with endogenous STX6 (**a**), GFP-RAB11 (**b**), GFP-RAB7 (**c**) and endogenous LAMTOR4 (**d**). Scale bars, 10 μm in top images and 5 μm in bottom axon images. **a-d**, left graphs, Quantification of colocalization between RUSH-LAMP1 and STX6 (a, 1h n=17, N=3; 4h n=16, N=3), RAB11 (b, 1h n=19, N=3; 4h n=18, N=3), RAB7 (c, 1h n=13, N=3; 4h n=14, N=3) and LAMTOR4 (d, 1h n=13, N=2; 4h n=19, N=3) in the soma by Mander's coefficients. Fraction of RUSH-LAMP1 on STX6/RAB11/RAB7/LAMTOR4 and fraction of STX6/RAB11/RAB7/LAMTOR4 on RUSH-LAMP1 are shown. **a-d**, right graphs, Quantification of colocalization between RUSH-LAMP1 and STX6 (a, 1h n=15, N=3; 4h n=15, N=3), RAB11 (b, 1h n=15, N=3; 4h n=18, N=3), RAB7 (c, 1h n=11, N=3; 4h n=14, N=3), LAMTOR4 (d, 1h n=17, N=3; 4h n=16, N=3) in the axon. Data are presented as mean values ± SD. ns–not significant, *p<0.05; ** p<0,01; *** p<0.001; **** p<0.0001. For Mander's analysis in the soma, Mann-Whitney test was used for fraction of RAB11/LAMTOR4 on RUSH and RUSH on RAB11/RAB7, unpaired t-test was used for STX6/RAB7 on RUSH and RUSH on STX6/LAMTOR4. For axonal colocalization analysis, unpaired t-test was used for endo STX6, RAB11, RAB7 and endo LAMTOR4. All experiments are repeated with indicated number of total number of neurons (n) and number of independent experiments (N). Source data are provided as a Source Data file (See Source Data 2).

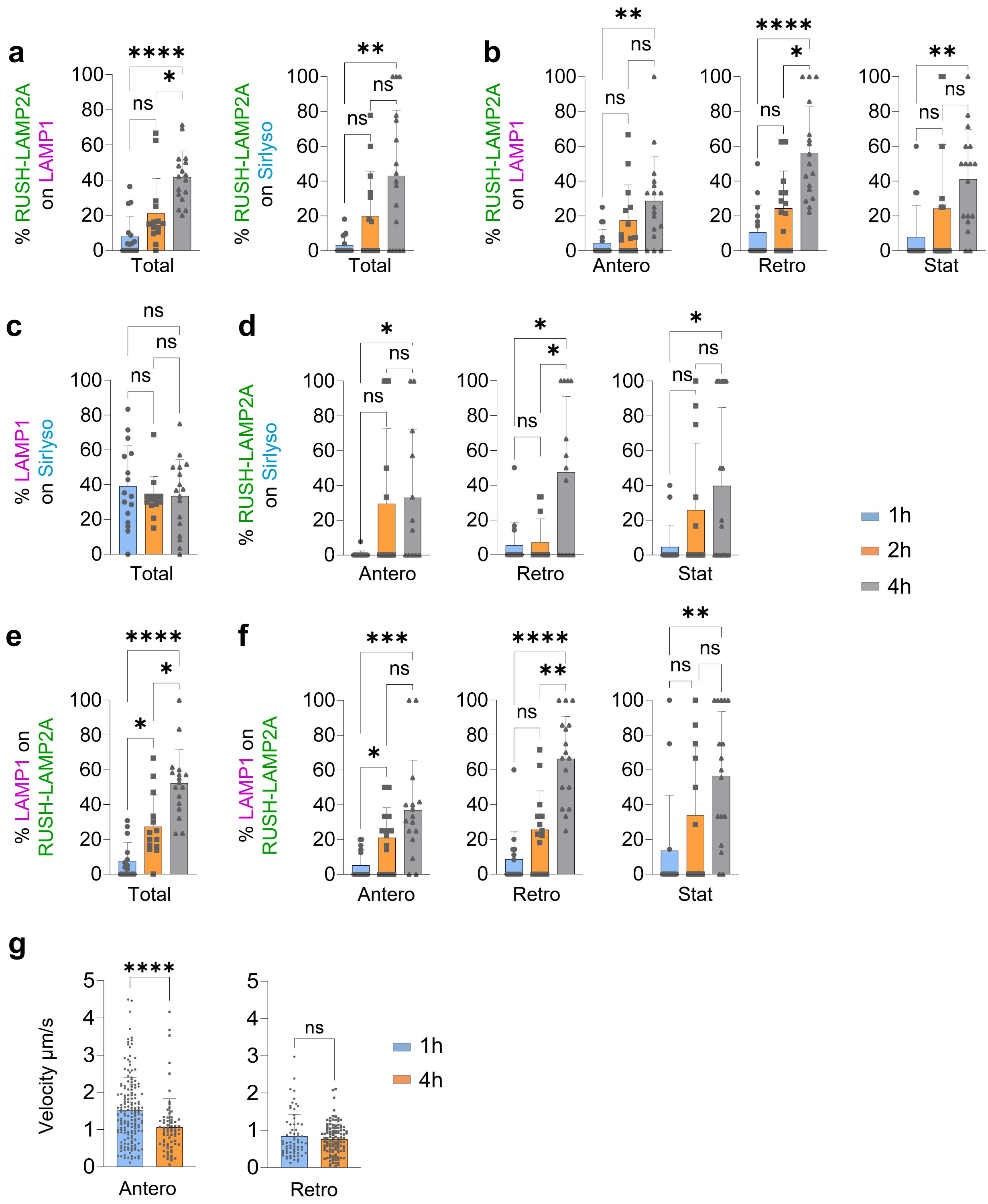

a
% RUSH-LAMP2A on LAMP1
% RUSH-LAMP2A on Sirlyso
Total
b
Antero
Retro
Stat
c
% LAMP1 on Sirlyso
d
e
% LAMP1 on RUSH-LAMP2A
f
g
Velocity µm/s
1h
2h
4h
ns
****
***
**
*

Supplementary Fig. 5 – Related to Fig. 4: Axonal biosynthetic LAMP trafficking dynamics with lysosomes over time.

a-f, Quantification of colocalized trajectories between RUSH-LAMP2A and LAMP1 (a, b); LAMP1 and SirLyso (c, d) or LAMP1 and RUSH-LAMP2A (e, f), after 1, 2 and 4h of biotin addition. n=16, 14 and 17 neurons, respectively; N=3 independent experiments. Total number of colocalizing compartments (a, c, e) and antero-, retrograde and stationary compartments (b, d, f). Data are presented as mean values. Kruskal-Wallis test followed by a Dunn's multiple comparison test. **g,** Velocity of anterograde and retrograde RUSH-LAMP2A after 1 and 4h of biotin addition. 1h n=16 neurons (Anterograde = 166 tracks; Retrograde = 68 tracks) and 4h n=17 neurons (Anterograde = 71 tracks; Retrograde = 113) respectively; N=3 independent experiments. Data are presented as mean values± SD, Mann-Whitney test, ns–not significant, **** $p<0.0001$. Source data are provided as a Source Data file (See Source Data 2).

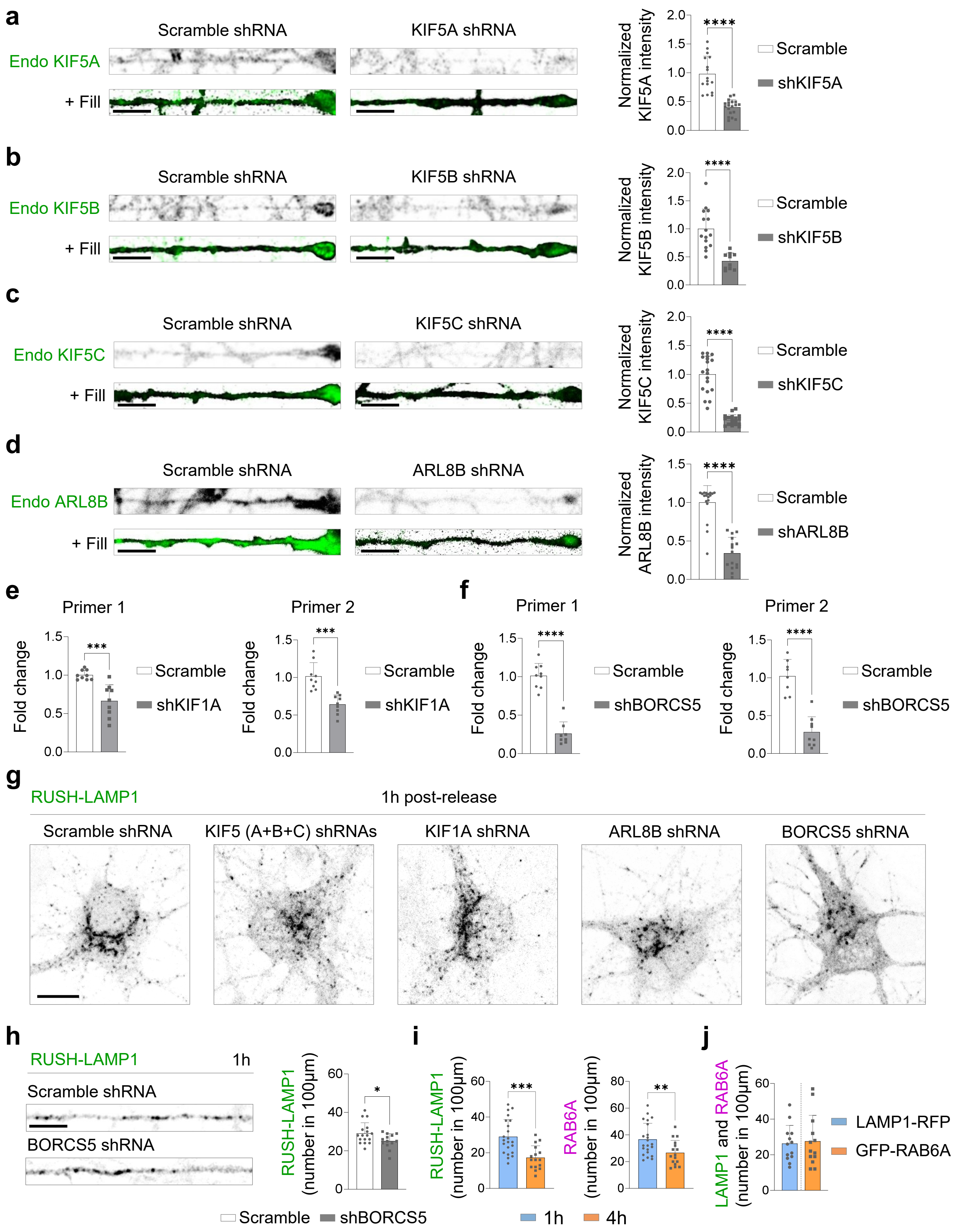
a
Scramble shRNA
KIF5A shRNA
Endo KIF5A
+ Fill
Normalized KIF5A intensity
****
Scramble
shKIF5A
b
Scramble shRNA
KIF5B shRNA
Endo KIF5B
+ Fill
Normalized KIF5B intensity
****
Scramble
shKIF5B
c
Scramble shRNA
KIF5C shRNA
Endo KIF5C
+ Fill
Normalized KIF5C intensity
****
Scramble
shKIF5C
d
Scramble shRNA
ARL8B shRNA
Endo ARL8B
+ Fill
Normalized ARL8B intensity
****
Scramble
shARL8B
e
Primer 1
Primer 2
Fold change
***
Scramble
shKIF1A
f
Primer 1
Primer 2
Fold change
****
Scramble
shBORCS5
g
RUSH-LAMP1
1h post-release
Scramble shRNA
KIF5 (A+B+C) shRNAs
KIF1A shRNA
ARL8B shRNA
BORCS5 shRNA
h
RUSH-LAMP1
1h
Scramble shRNA
BORCS5 shRNA
RUSH-LAMP1 (number in 100µm)
*
Scramble
shBORCS5
i
RUSH-LAMP1 (number in 100µm)
***
RAB6A (number in 100µm)
**
1h
4h
j
LAMP1 and RAB6A (number in 100µm)
LAMP1-RFP
GFP-RAB6A

Supplementary Fig. 6 – Related to Fig. 5: BORCS5 depletion on biosynthetic LAMP trafficking in the axon and biosynthetic LAMP characterization with RAB6A.

a-d, Confocal images of axon tips from neurons expressing scramble shRNA and shRNA against KIF5A/B/C and ARL8B immunostained for endogenous KIF5A (a, scramble n=16; shKIF5A n=19; N=3 independent experiments), endogenous KIF5B (b, scramble n=16; shKIF5B n=10; N=3 independent experiments), endogenous KIF5C (c, scramble n=19, shKIF5C n=18; ; N=3 independent experiments), and endogenous ARL8B (d, scramble n=18, shARL8B n=15; N=3 independent experiments). Knockdown efficiency was quantified by normalizing knockdown intensity to scramble control intensity along axon tips. **e-f,** Quantitative PCR fold change of shKIF1A (e) and shBORCS5 (f) to scramble control using 2 different primer pairs. All values were normalized to respective GAPDH internal control. N=3 independent experiments with 3 technical replicates per biological sample. **g,** Confocal images of somas from neurons expressing RUSH-LAMP1-V5 and shRNAs: scramble, KIF5(A+B+C), KIF1A, ARL8B and BORCS5. **h**, Confocal images of axons of neurons expressing RUSH-LAMP1-V5 and scramble or BORCS5 shRNA. Quantification of RUSH-LAMP1-V5 vesicles in 100 µm axon (scramble n=17, shBORCS5 n=13; N=3 independent experiments). **i,** Number of RUSH-LAMP1-V5 and RAB6A compartments in 100 µm axon at 1h and 4h after release. n=22 and 16 neurons, respectively; N=3 independent experiments. **j**, Total number of LAMP1 and RAB6A compartments at the steady state. n=13, N=4 independent experiments. Scale bars, 5 µm in (a), (b), (c), (d), (h), and 10 µm in (g). Data are presented as mean values ± SD, plus individual points. * p<0.05; ** p<0,01; *** p<0.001, **** p<0.0001. Unpaired t-test was used in (a), (b), (c), (e), (f, Primer 2), (h), (i), and (j) and Mann-Whitney test in (d) and (f, Primer 1). Representative images were repeated in at least 3 independent experiments. Source data are provided as a Source Data file (See Source Data 2).

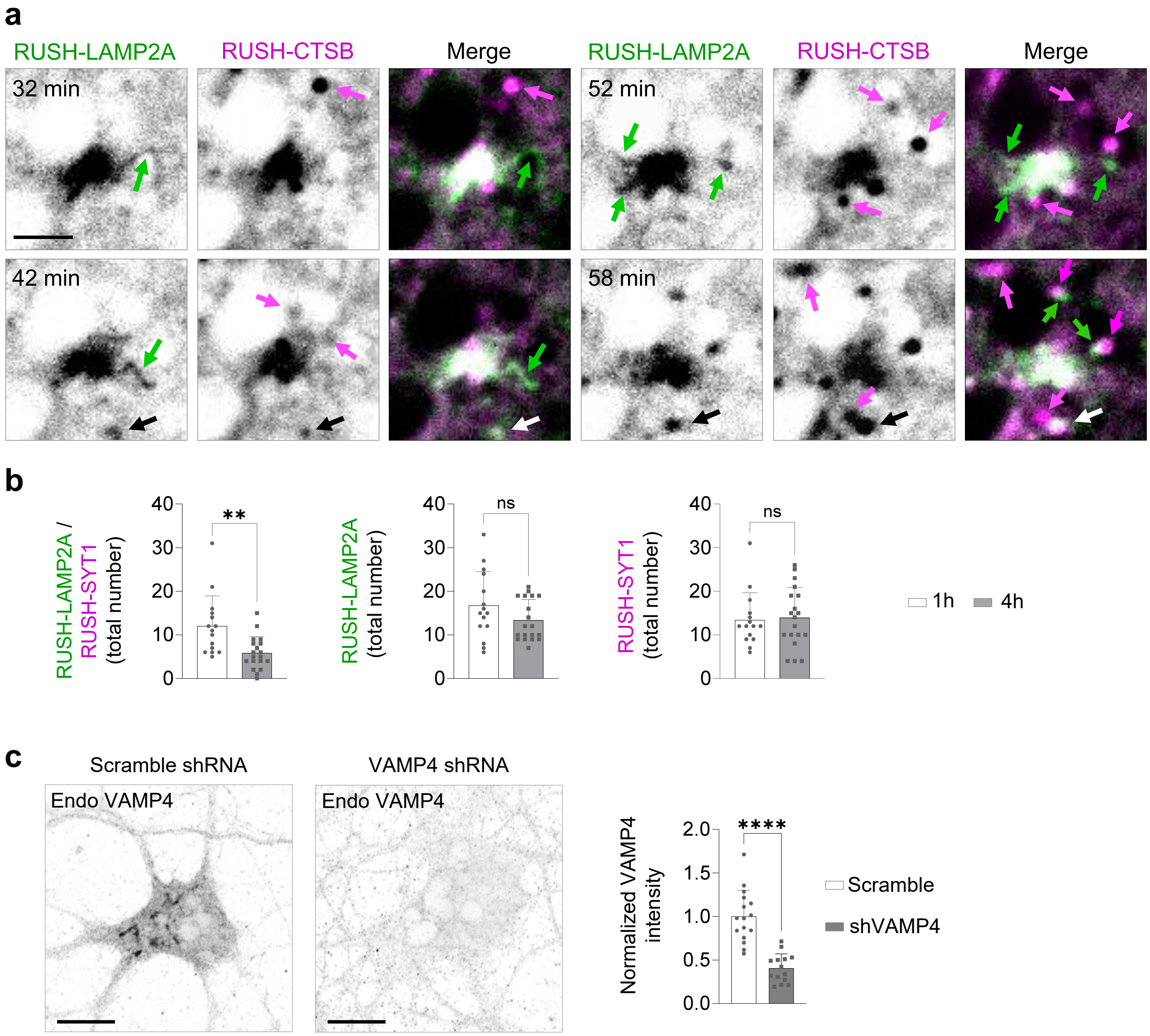

a
RUSH-LAMP2A
RUSH-CTSB
Merge
RUSH-LAMP2A
RUSH-CTSB
Merge
32 min
52 min
42 min
58 min
b
RUSH-LAMP2A / RUSH-SYT1 (total number)
RUSH-LAMP2A (total number)
RUSH-SYT1 (total number)
**
ns
ns
1h
4h
c
Scramble shRNA
VAMP4 shRNA
Endo VAMP4
Endo VAMP4
Normalized VAMP4 intensity
****
Scramble
shVAMP4

Supplementary Fig. 7 – Related to Fig. 6: Biosynthetic LAMP and CTSB exits in distinct compartments from the TGN.

a, Still images of a region of the soma from a live neuron expressing RUSH-LAMP2A-mNG and RUSH-CTSB-Halo. Compartments budding from the Golgi are indicated with green arrows for RUSH-LAMP2A-mNG and magenta arrows for RUSH-CTSB-Halo. White arrows despite a post-Golgi fusion event. **b**, Neurons expressing RUSH-LAMP2A-mNG and RUSH-SYT1-Halo at 1 and 4h post-release. Total number of RUSH-LAMP1-V5 and RUSH-SYT1 compartments at 1h or 4h post-release. Colocalizing compartments to the left; total RUSH-LAMP1-V5 and RUSH-SYT1 in the middle and on the right, respectively. n=15 and 19 neurons; N=3 independent experiments. **c**, Immunostaining of endogenous VAMP4 in neurons transfected with scramble shRNA or shRNA targeting VAMP4 and quantification of the intensity of VAMP4 signal in the soma in VAMP4 KD cells versus scramble. Scramble n=16, shVAMP4 n=14, N=3 independent experiments. Scale bars, 2 µm in (a), and 10 µm in (c). Data are presented as mean values ± SD, plus individual points. ns–not significant, ** p<0,01, ****p<0.0001. Mann-Whitney test in (b), and unpaired *t*-test in (c). Representative images were repeated in at least 3 independent experiments. Source data are provided as a Source Data file (See Source Data 2).

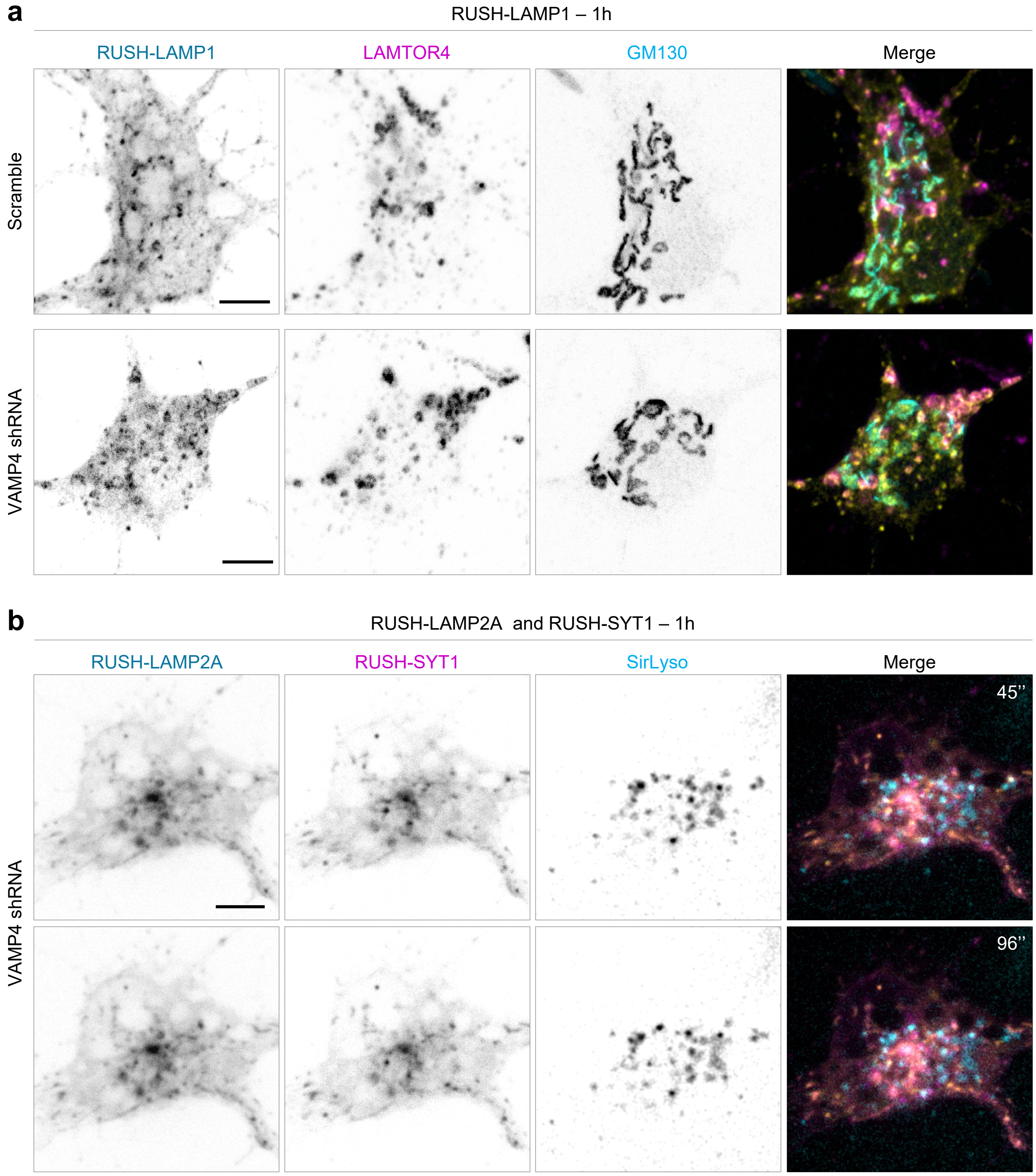
a
RUSH-LAMP1 – 1h
RUSH-LAMP1
LAMTOR4
GM130
Merge
Scramble
VAMP4 shRNA
b
RUSH-LAMP2A and RUSH-SYT1 – 1h
RUSH-LAMP2A
RUSH-SYT1
SirLyso
Merge
45''
96''
VAMP4 shRNA

Supplementary Fig. 8 – Related to Fig. 6: VAMP4 depletion does not alter Golgi morphology or biosynthetic cargo release.

a, Confocal images of somas from neurons expressing RUSH-LAMP1-V5 and scramble or VAMP4 shRNA, fixed 1h after release and stained for LAMTOR4 and GM130. **c**, Still images of the soma of a live neuron expressing RUSH-LAMP2A-mNG and RUSH-SYT1-Halo, and VAMP4 shRNA, 1h after release and labeled with SirLyso. Scale bar, 5 μm in (a) and (b). Representative images were repeated in at least 3 independent experiments.

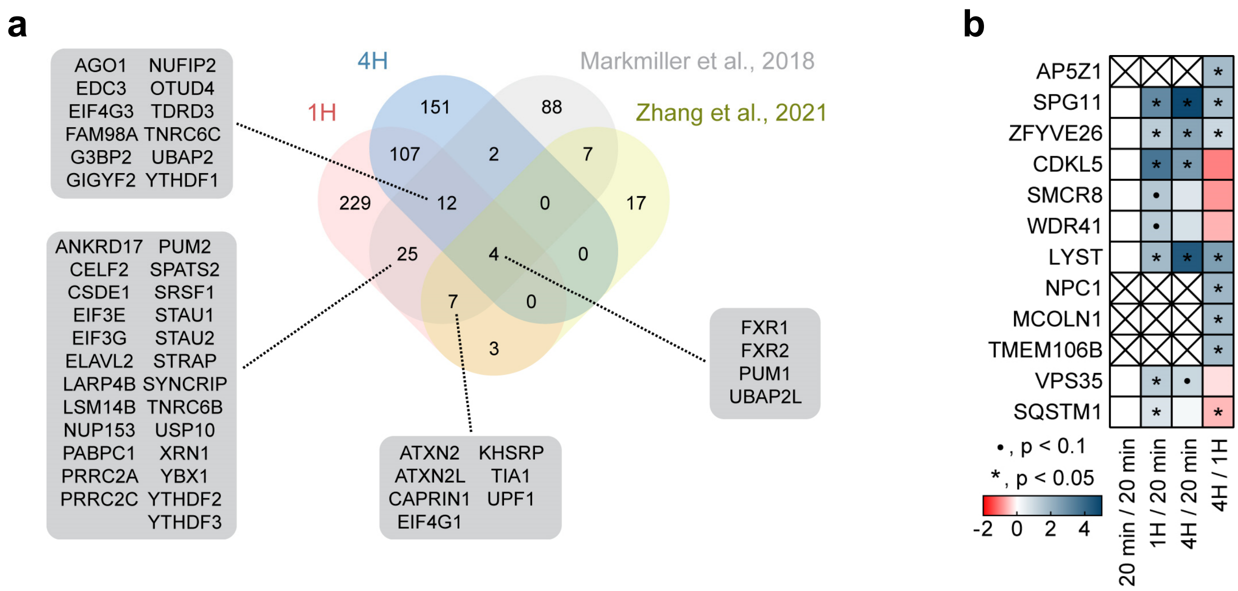
a
4H
Markmiller et al., 2018
1H
Zhang et al., 2021
151
88
107
2
7
229
12
0
17
25
4
0
7
0
3
AGO1 NUFIP2
EDC3 OTUD4
EIF4G3 TDRD3
FAM98A TNRC6C
G3BP2 UBAP2
GIGYF2 YTHDF1
ANKRD17 PUM2
CELF2 SPATS2
CSDE1 SRSF1
EIF3E STAU1
EIF3G STAU2
ELAVL2 STRAP
LARP4B SYNCRIP
LSM14B TNRC6B
NUP153 USP10
PABPC1 XRN1
PRRC2A YBX1
PRRC2C YTHDF2
YTHDF3
ATXN2 KHSRP
ATXN2L TIA1
CAPRIN1 UPF1
EIF4G1
FXR1
FXR2
PUM1
UBAP2L
b
AP5Z1
SPG11
ZFYVE26
CDKL5
SMCR8
WDR41
LYST
NPC1
MCOLN1
TMEM106B
VPS35
SQSTM1
•, p < 0.1
*, p < 0.05
-2 0 2 4
20 min / 20 min
1H / 20 min
4H / 20 min
4H / 1H

Supplementary Fig. 9 – Related to Fig. 7: POTATOMap identifies RNA granule proteins and lysosome related disease proteins over time.

a, Venn diagram of proteins identified in the 1h and 4h time points compared to datasets from Markmiller et al., 2018 and Zhang et al., 2021 curated from RNA granule database[73,74,75]. Shared proteins were highlighted. **b**, Heatmap of highlighted disease-related proteins found in POTATOMap screen with *p* value range indicated (two-sided t-test, no multiple comparison test was used). Source data are provided as a Source Data file (See Source Data 1).
