# Supplementary material for: Spatiotemporal proteomics reveals the biosynthetic lysosomal membrane protein interactome in neurons
Source: Nat Commun. 2024 Dec 30;15:10829. doi: 10.1038/s41467-024-55052-w (PMC11868546; doi:10.1038/s41467-024-55052-w)
Supplement: Supplementary file 2 — Reporting Summary [file 41467_2024_55052_MOESM2_ESM.pdf]

nature portfolio

Corresponding author(s): Ginny G. Farias

Last updated by author(s): Sep 23, 2024

Reporting Summary

Nature Portfolio wishes to improve the reproducibility of the work that we publish. This form provides structure for consistency and transparency in reporting. For further information on Nature Portfolio policies, see our Editorial Policies and the Editorial Policy Checklist.

Statistics

For all statistical analyses, confirm that the following items are present in the figure legend, table legend, main text, or Methods section.

n/a	Confirmed	
☐	☒	The exact sample size (*n*) for each experimental group/condition, given as a discrete number and unit of measurement
☐	☒	A statement on whether measurements were taken from distinct samples or whether the same sample was measured repeatedly
☐	☒	The statistical test(s) used AND whether they are one- or two-sided<br>*Only common tests should be described solely by name; describe more complex techniques in the Methods section.*
☐	☒	A description of all covariates tested
☐	☒	A description of any assumptions or corrections, such as tests of normality and adjustment for multiple comparisons
☐	☒	A full description of the statistical parameters including central tendency (e.g. means) or other basic estimates (e.g. regression coefficient) AND variation (e.g. standard deviation) or associated estimates of uncertainty (e.g. confidence intervals)
☐	☒	For null hypothesis testing, the test statistic (e.g. *F*, *t*, *r*) with confidence intervals, effect sizes, degrees of freedom and *P* value noted<br>*Give P values as exact values whenever suitable.*
☒	☐	For Bayesian analysis, information on the choice of priors and Markov chain Monte Carlo settings
☒	☐	For hierarchical and complex designs, identification of the appropriate level for tests and full reporting of outcomes
☒	☐	Estimates of effect sizes (e.g. Cohen's *d*, Pearson's *r*), indicating how they were calculated

Our web collection on statistics for biologists contains articles on many of the points above.

Software and code

Policy information about availability of computer code

Data collection	All fixed cell imaging was performed using a Zeiss LSM 900 confocal microscope equipped with Zen imaging software (Zeiss) Zen Blue software version 3.7.97.07000. All live imaging experiments were performed using a i Nikon Eclipse Ti-E inverted microscope equipped with MetaMorph (Molecular Devices) version 7.10.2.240 for controlling all devices. Western blots were exposed and imaged using an Odyssey Clx imaging system (LICOR) with Image Studio version 5.2 software and ImageQuant 800 (AMERSHAM). Proteomic raw data was analyzed and collected on a Orbitrap Exploris 480 mass spectrometer (Thermo Fisher Scientific) coupled to an UltiMate 3000 UHPLC system (Thermo Fisher Scientific).
Data analysis	Database search of the proteomics raw data was conducted using MaxQuant (v2.0.1.0). Further analysis and statistical test was performed using Perseus (v1.6.15.0) and online STRING database (v12.0). Image processing and analysis was performed using ImageJ 1x/FIJI software. Microsoft Excel was used for data collection and Graphpad Prism 9 software was used to perform statistical analysis and data illustration. No custom codes were used in this study.

For manuscripts utilizing custom algorithms or software that are central to the research but not yet described in published literature, software must be made available to editors and reviewers. We strongly encourage code deposition in a community repository (e.g. GitHub). See the Nature Portfolio guidelines for submitting code & software for further information.

Data

Policy information about availability of data

All manuscripts must include a data availability statement. This statement should provide the following information, where applicable:

- Accession codes, unique identifiers, or web links for publicly available datasets
- A description of any restrictions on data availability
- For clinical datasets or third party data, please ensure that the statement adheres to our policy

Datasets generated during and/or analyzed during the current study are available in Source Data of the manuscript. Proteomics dataset will be deposited on PRIDE.

Research involving human participants, their data, or biological material

Policy information about studies with human participants or human data. See also policy information about sex, gender (identity/presentation), and sexual orientation and race, ethnicity and racism.

Reporting on sex and gender	N/A
Reporting on race, ethnicity, or other socially relevant groupings	N/A
Population characteristics	N/A
Recruitment	N/A
Ethics oversight	N/A

Note that full information on the approval of the study protocol must also be provided in the manuscript.

Field-specific reporting

Please select the one below that is the best fit for your research. If you are not sure, read the appropriate sections before making your selection.

☒ Life sciences ☐ Behavioural & social sciences ☐ Ecological, evolutionary & environmental sciences

For a reference copy of the document with all sections, see nature.com/documents/nr-reporting-summary-flat.pdf

Life sciences study design

All studies must disclose on these points even when the disclosure is negative.

Sample size	Sample sizes were not pre-determined. We chose samples sizes based on previously observed variation in imaging of our primary cultures of neurons. Because of the nature of primary neuron cultures, neurons might have variation in their growth rate, morphology, or expression levels. To eliminate the effects from these variations, we used 10 to 30 neurons per quantifications from fixed cells' imaging and at least 14 neurons for quantifications from live cell imaging. Similar sample sizes have been reported in other publications (Farias et al., 2015; Tortosa et al., 2017; Farias et al., 2017; Farias et al 2019; Pan et al., 2019; De Pace et al., 2020)
Data exclusions	No data were excluded from analyses
Replication	All imaging experiments were repeated from at least 2 to 3 biological replicates. Representative images were shown. Due to sample limitation, proteomics data were collected from 2 biological replicates and each replicate were analyzed with 2 technical replicates.
Randomization	Randomization is not relevant for our experiments as samples were not allocated into groups.
Blinding	Investigators were not blinded for data collection and data analysis. Since most of the experimental procedures and data analyses were performed by more than one researcher obtaining similar results and all the data analysis were performed by using softwares, blinding was not relevant for this study.

Reporting for specific materials, systems and methods

We require information from authors about some types of materials, experimental systems and methods used in many studies. Here, indicate whether each material, system or method listed is relevant to your study. If you are not sure if a list item applies to your research, read the appropriate section before selecting a response.

Materials & experimental systems

n/a	Involved in the study
	[x] Antibodies
	[x] Eukaryotic cell lines
[x]	Palaeontology and archaeology
	[x] Animals and other organisms
[x]	Clinical data
[x]	Dual use research of concern
[x]	Plants

Methods

n/a	Involved in the study
[x]	ChIP-seq
[x]	Flow cytometry
[x]	MRI-based neuroimaging

Antibodies

Antibodies used

rabbit anti-LAMTOR4 (Cell Signaling, clone D6A4V, Cat# 12284S, RRID: AB_2797870, 1/500),
mouse anti-STX6 (BD Biosciences Cat# 610635, RRID:AB_397965, 1/100),
rabbit anti-VAMP4 (Synaptic Systems, Cat# 136002, RRID:AB_887816, 1/100),
mouse anti-V5 (Thermo Fisher Scientific Cat# R960-25, RRID:AB_2556564, 1/1000 for IF and WB),
mouse anti-Pan-Neurofascin external (clone A12/18; UC Davis/NIH NeuroMab, Cat# 75-172, RRID: AB_2282826, 0.18mg/ml),
in-house rabbit anti-TRIM46 (1/1000),
mouse anti-VTI1B (BD Biosciences Cat# 611404, RRID:AB_398926, 1/250),
rabbit anti-GM130 (Abcam Cat# ab52649, RRID:AB_880266, 1/800),
rabbit anti-ARL8B (Proteintech Cat# 13049-1-AP, RRID:AB_2059000, 1/500),
rabbit anti-KIF5A (Abcam Cat# ab5628, RRID:AB_2132218, 1/100),
rabbit anti-KIF5B (Abcam Cat# ab5629, RRID:AB_2132379, 1/100),
rabbit anti-KIF5C (Abcam Cat# ab5630, 1/100),
rabbit anti-mouse immunoglobulins/HRP (Agilent Cat# P0260, RRID:AB_2636929, 1/10000),
goat anti-rabbit IgG (H+L) Highly cross-absorbed secondary antibody Alexa Fluor 405 (Thermo Fisher Scientific Cat# A-31556, RRID:AB_221605, 1/1000),
donkey anti-rabbit IgG (H+L) Highly cross-absorbed secondary antibody Alexa Fluor 647 (Thermo Fisher Scientific Cat# A-31573, RRID:AB_2536183, 1/1000),
goat anti-rabbit IgG (H+L) highly cross-absorbed secondary antibody Alexa Fluor 568 (Thermo Fisher Scientific Cat# A-11036, RRID:AB_10563566, 1/1000),
goat anti-mouse IgG1 cross-absorbed secondary antibody Alexa Fluor 594 (Thermo Fisher Scientific Cat# A-21125, RRID:AB_2535767, 1/1000),
goat anti-mouse IgG2a cross-absorbed secondary antibody Alexa Fluor 594 (Thermo Fisher Scientific Cat# A-21135, RRID:AB_2535774, 1/1000),

Validation

Rabbit anti-LAMTOR4 (Cell Signaling, clone D6A4V, Cat# 12284S, RRID: AB_2797870): manufacturer’s website noted that LAMTOR4/C7orf59 (D6A4V) Rabbit mAb recognizes endogenous levels of total LAMTOR4/C7orf59 protein and has reactivity for human, mouse, rat and monkey. Some of the relevant citations are Jia R et al., 2019; Janssen AF et al., 2018; Sun J et al., 2018, Özkan et al., 2021. Data provided in manuscript in Figure 3e, S4d, S8.
Mouse anti-STX6 (BD Biosciences Cat# 610635, RRID:AB_397965): manufacturer's website noted that STX6 recognizes endogenous levels of total STX6 protein from immunogen aa 6-136. Antibody is tested against human, mouse, rat, dog, chicken and frog. Relevant citations include Andres-Alonso M et al., 2023; Kanatsu K et al., 2018; Tie et al., 2018. Data provided in manuscript in Figure 3b and supplementary figure 4a.
Rabbit anti-VAMP4 (Synaptic Systems, Cat# 136002, RRID:AB_887816): manufacturer's website noted that VAMP4 antibody is K.O. verified, targeting rat VAMP4 aa 1-117. Relevant citation include Bremner et al., 2023; Bakr et al., 2021. Data provided in manuscript in supplementary figure 7c also denotes antibody specificity from knockdown experiments.
Mouse anti-V5 (Thermo Fisher Scientific Cat# R960-25, RRID:AB_2556564): manufacturer's website noted that V5 mouse mAb recognizes V5 synthetic peptide Gly-Lys-Pro-Ile-Pro-Asn-Pro-Leu-Leu-Gly-Leu-Asp-Ser-Thr. Relevant citations include Han et al., 2017; Qin et al., 2023. Data provided in manuscript in Figure 2c,d, S2g, 3b-e, S4a-d, 5b,d,e,f, S6g,h, 6e,f, 7b.
Mouse anti-Pan-Neurofascin external (clone A12/18; UC Davis/NIH NeuroMab, Cat# 75-172, RRID: AB_2282826): manufacturer’s website noted that antibody was tested for immunoblotting, immunohistochemistry and has reactivity for human and rat.
rabbit anti-TRIM46 (Homemade; validated in van Beuningen et al., 2015). Antibody has been used in several publications, including Tortosa et al., 2017; Farias et al., 2019; Pan et al., 2019; Harterink et al., 2019; Freal et al., 2019; Lindhout et al., 2021.
Mouse anti-VTI1B (BD Biosciences Cat# 611404, RRID:AB_398926): manufacturer's website provided that VTI1B targets human, mouse, rat VTI1B at aa9-121. Relevant citations include Li et al., 2019. Data provided in manuscript in Figure S2g.
Rabbit anti-GM130 (Abcam Cat# ab52649, RRID:AB_880266): manufacturer's website provided that monoclonal anti-GM130 recognizes human, dog and african green monkey GM130. Relevant citations include Abudu et al., 2024; Byappanahalli et al., 2024. Data provided in this manuscript in Figure S8.
Rabbit anti-ARL8B (Proteintech Cat# 13049-1-AP, RRID:AB_2059000): manufacturer's website provided that Rabbit anti-ARL8B recognizes human, mouse, rat, monkey and chicken ARL8B. Relevant citations include Hummel et al., 2021; Menon et al., 2023, Schleinitz et al., 2023. Data provided in the manuscript in Figure S6d.
Rabbit anti-KIF5A (Abcam Cat# ab5628, RRID:AB_2132218): manufacturer's website provided that Rabbit anti-KIF5A recognizes human, mouse, rat and cow KIF5A. Relevant citations include Shi et al., 2018; Furey et al., 2020. Data provided in the manuscript in Figure S6a.
Rabbit anti-KIF5B (Abcam Cat# ab5629, RRID:AB_2132379): manufacturer's website provided that Rabbit anti-KIF5B recognizes

human, mouse and rat KIF5B. Relevant citations include Lazo et al., 2023. Data provided in the manuscript in Figure S6b. Rabbit anti-KIF5C (Abcam Cat# ab5630): manufacturer's website provided that Rabbit anti-KIF5C recognizes human KIF5C. from aa900 to C terminus. Relevant citations include Swarnkar et al., 2024; Pegg et al., 2021. Data provided in the manuscript in Figure S6c.

Eukaryotic cell lines

Policy information about cell lines and Sex and Gender in Research

Cell line source(s)	HEK293T cell line from ATCC. Rat INS-1 insulinoma cell line, CLone 832/3 (SCC208), Merck. INS-1 832/3 is a derivative of INS-1 originally established from an x-ray induced insulinoma in rat. The INS-1 832/3 cell line is a subclone of INS-1 that was stably transfected with a CMV promoter-human insulin expression plasmid carrying a geneticin (G418)-resistance marker for selection.
Authentication	HEK293T cells were verified by ATCC and INS-1 were verified by Merck.
Mycoplasma contamination	HEK293T and INS-1 cell lines were tested negative for mycoplasma
Commonly misidentified lines (See ICLAC register)	Misidentified lines were not used in this study.

Animals and other research organisms

Policy information about studies involving animals; ARRIVE guidelines recommended for reporting animal research, and Sex and Gender in Research

Laboratory animals	2.5 months old female pregnant Wistar rats were obtained from Janvier, and embryos (both genders) at E18 stage of development were used for primary cultures of hippocampal and cortical neurons. The animals, pregnant females and embryos have not been involved in other procedures.
Wild animals	This study did not involve wild animals
Reporting on sex	This study did not involve gender specific experiments on animals.
Field-collected samples	This study did not involve sample collected from fields.
Ethics oversight	All experiments were approved by the DEC Dutch Animal Experiments Committee (Dier Experimenten Commissie), performed in line with institutional guidelines of University Utrecht and conducted in agreement with Dutch law (Wet op de Dierproeven, 1996) and European regulations (Directive 2010/63/EU). The animal protocol has been evaluated and approved by the national CCD authority (license AVD10800202216383). Information is provided in the manuscript.

Note that full information on the approval of the study protocol must also be provided in the manuscript.

Plants

Seed stocks	N/A
Novel plant genotypes	N/A
Authentication	N/A
